# Acidic Pre-Conditioning Enhances the Stem Cell Phenotype of Human Bone Marrow Stem/Progenitor Cells

**DOI:** 10.3390/ijms20051097

**Published:** 2019-03-04

**Authors:** Yuri Hazehara-Kunitomo, Emilio Satoshi Hara, Mitsuaki Ono, Kyaw Thu Aung, Keiko Komi, Hai Thanh Pham, Kentaro Akiyama, Masahiro Okada, Toshitaka Oohashi, Takuya Matsumoto, Takuo Kuboki

**Affiliations:** 1 Department of Oral Rehabilitation and Regenerative Medicine, Okayama University Graduate School of Medicine, Dentistry and Pharmaceutical Sciences, Okayama 700-8525, Japan; de19034@s.okayama-u.ac.jp (Y.H.-K.); kyawthu80@gmail.com (K.T.A.); de17026@s.okayama-u.ac.jp (K.K.); pthai@hpmu.edu.vn (H.T.P.); akentaro@md.okayama-u.ac.jp (K.A.); 2 Department of Biomaterials, Okayama University Graduate School of Medicine, Dentistry and Pharmaceutical Sciences, Okayama 700-8525, Japan; m_okada@cc.okayama-u.ac.jp (M.O.); tmatsu@md.okayama-u.ac.jp (T.M.); 3 Department of Molecular Biology and Biochemistry, Okayama University Graduate School of Medicine, Dentistry and Pharmaceutical Sciences, Okayama 700-8558, Japan; oohashi@cc.okayama-u.ac.jp; 4 Craniofacial and Skeletal Diseases Branch, NIDCR, NIH, Bethesda, MD 20892, USA; 5 Department of Oral Surgery, Faculty of Dentistry, Haiphong University of Medicine and Pharmacy, Haiphong 04212, Vietnam

**Keywords:** stemness, mesenchymal stem cells, acidic treatment, bone healing

## Abstract

A deeper understanding of the detailed mechanism of in vivo tissue healing is necessary for the development of novel regenerative therapies. Among several external factors, environmental pH is one of the crucial parameters that greatly affects enzyme activity and cellular biochemical reactions involving tissue repair and homeostasis. In this study, in order to analyze the microenvironmental conditions during bone healing, we first measured the pH in vivo at the bone healing site using a high-resolution fiber optic pH microsensor directly in femur defects and tooth extraction sockets. The pH was shown to decrease from physiological 7.4 to 6.8 during the initial two days of healing (inflammatory phase). In the same initial stages of the inflammatory phase of the bone healing process, mesenchymal stem cells (MSCs) are known to migrate to the healing site to contribute to tissue repair. Therefore, we investigated the effect of a short-term acidic (pH 6.8) pre-treatment on the stemness of bone marrow-derived MSCs (BMSCs). Interestingly, the results showed that pre-treatment of BMSCs with acidic pH enhances the expression of stem cell markers (OCT-4, NANOG, SSEA-4), as well as cell viability and proliferation. On the other hand, acidic pH decreased BMSC migration ability. These results indicate that acidic pH during the initial stages of bone healing is important to enhance the stem cell properties of BMSCs. These findings may enable the development of novel methods for optimization of stem cell function towards tissue engineering or regenerative medicine.

## 1. Introduction

Bone healing is known to involve an acute inflammatory period, followed by the recruitment of mesenchymal stem cells (MSCs), revascularization, and bone remodeling [1]. During the inflammation phase, which has its peak in the initial 48 h of wound healing, chemokines and pro-inflammatory cytokines, such as interleukins (e.g., IL-1β, IL-6) and tumor necrosis factor-α (TNF-α), are released by activated macrophages and initiate the inflammatory cascade, which is important not only for recruiting leucocytes, but also for recruitment and activation of surrounding connective tissue cells, including stem/progenitor cells [2]. Previous reports have demonstrated that MSCs migrate to the wound site during the initial two days of healing, at the inflammatory phase, and contribute to tissue healing [1,2,3,4,5]. However, the environmental condition is known as an important factor affecting the functions of stem cells, such as cell migration, proliferation, and stemness or differentiation ability. Therefore, further understanding of the detailed process of in vivo bone healing as well as of the biophysical and biochemical changes in the microenvironment may enable the development of novel methods to control MSC function and/or to enhance bone regeneration.

In this context, ionic concentrations (e.g., pH) in the wound milieu directly affects the biochemical reactions associated with wound healing, including the optimal pKa values for enzymes. Indirectly, pH can also affect cellular signaling and promote or inhibit cellular functions, including secretion of cytokines and synthesis of extracellular matrix [6]. Despite the great number of reports describing pH levels during soft tissue healing, the literature regarding the measurement of local pH during bone healing is limited. A previous paper has indicated that the pH is neutral to slightly acidic on the second day of healing (inflammatory phase) [7]. The method used for pH measurement, however, was based on non-invasive nuclear magnetic resonance, which just gives an estimation of the pH at the healing site, and the study had not evaluated the changes in pH in the initial days of healing, which could possibly affect the function of stem cells.

Therefore, a more direct and robust measurement of pH at the healing site in vivo is required. Additionally, the effect of pH on mesenchymal stem cells has not been completely clarified. In this study, we first directly measured the pH variation during bone healing using an optical fiber-based pH microsensor and then analyzed the effect of a short-term acidic pH treatment on the regulation of stemness and function of human bone marrow stem/progenitor cells (hBMSCs).

## 2. Results

### 2.1. In Vivo Measurement of pH Variation during Bone Healing

**Figure 1:**
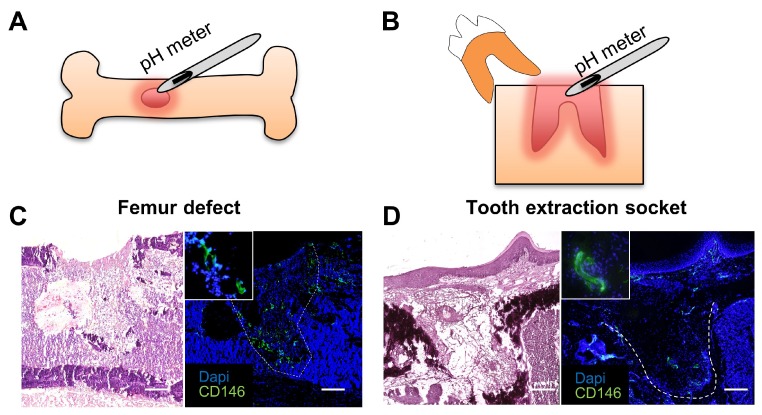
Models of bone defects in (**A**) mouse femur and (**B**) tooth extraction socket. Hematoxylin-Eosin staining (left) and immunostaining for the mesenchymal stem cell (MSC) marker CD146 (right) of (**C**) mouse femur defect and (**D**) tooth extraction socket. Note that CD146^+^ MSCs (including pericytes) migrate into the healing site in the initial days of the inflammatory period of tissue healing. Images are representative of at least three independent experiments. Histological images show the healing site on day 1 post-surgery. Scale bars: 200 μm.

Figure 1A,B show the models for analysis of bone healing in mouse femur (bone defect) and in the tooth extraction socket. Note that CD146^+^ MSCs migrate into the granulation tissue during the initial two days (inflammatory period) of bone healing (Figure 1C,D), which is in accordance with previous reports [2]. These MSCs were also shown to be present at the healing site until day 3, but could not be detected onwards from day 4 or 5 post-surgery [2,3].

**Figure 2:**
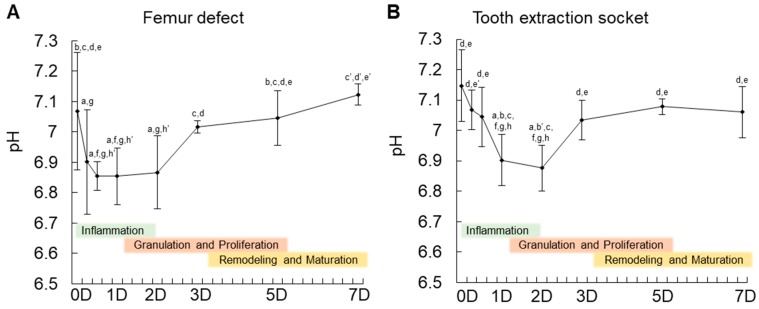
Measurement of pH levels during the initial 7 days of bone healing in (**A**) mouse femur defect and (**B**) tooth extraction socket. Note the rapid decrease in pH down to pH 6.8 in the initial 12 h of the inflammatory period. The error bars represent the standard deviation of the pH measurements from 4 different healing sites. The letters (a–h) show a statistically significant difference (*p* ≤ 0.05) compared to: a = 0 h, b = 6 h, c = 12 h, d = 1 day (1D), e = 2 days (2D), f = 3 days (3D), g = 5 days (5D), and h = 7 days (7D). The letters b’, c’, d’, e’ and h’ show a statistically significant difference (*p* ≤ 0.01) compared to: b’ = 6 h, c’ = 12 h, d’ = 1D, e’ = 2D, and h’ = 7D. Statistics were performed using one-way ANOVA and Fisher’s post-hoc tests.

Next, to obtain more insight into the environmental changes during healing of bone wounds, pH levels were measured using a high-resolution optical fiber-based pH microsensor during the seven days of healing before granulation tissue mineralization. As shown in Figure 2, pH at the granulation tissue of the bone defect decreased rapidly in the initial 6 h post-surgery, achieving the lowest level (pH 6.85) after 12 h. The pH maintained in acidic condition until day 2, and then increased slightly until day 3, and returned to normal levels after 7 days, with a tendency to extend toward an alkaline condition (Figure 2).

At the tooth extraction socket, the decrease in pH levels was slower in the initial hours, compared to the femur defect, but it also reached the same acidity (pH 6.85) at 12 h after tooth extraction. Similar to the pH levels in the femur defect, the pH levels increased slowly onwards from day 2, reaching a plateau at day 7 after tooth extraction.

These results indicate that the initial two days of the inflammatory period is acidic. We then investigated the effect of a short-term acidic (pH 6.8) treatment on the physiological function of MSCs.

### 2.2. Short-Term Acidic Stimulation Enhances the Viability and Proliferation of MSCs

**Figure 3:**
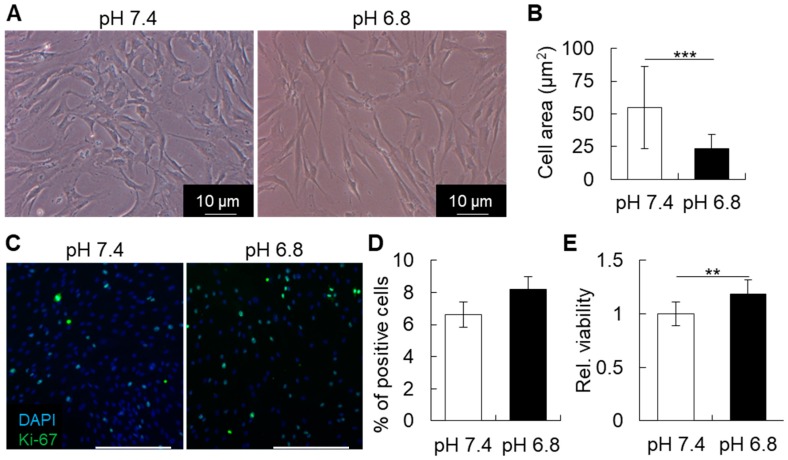
(**A**) Photographs of human bone marrow stem/progenitor cell (hBMSC) shape during culture in control (physiologic pH 7.4) or acidic pH (pH 6.8). (**B**) Quantitative analysis of cell area, demonstrating the change in cell shape in a slightly acidic condition. (**C**,**D**) Immunostaining and quantitative analysis of actively proliferating Ki-67^+^ hBMSCs. Scale bars: 500 μm. There was an increase in the number of cells positive for Ki-67, but statistically, the difference was not significant. (**E**) The graph shows the quantitative analysis of cell viability estimated by MTS assay. The results suggest that a short-term treatment with pH 6.8 enhances the viability of hBMSCs. ** *p* ≤ 0.01, *** *p* ≤ 0.001; Student’s *t*-test.

In order to evaluate the changes in the morphology and viability of hBMSCs, the cells were cultured in acidic pH for two days and then assayed. As shown in Figure 3A,B, the cells became slightly thinner after acidic stimulation, compared to control (pH 7.4) group. The MTS assay, however, suggested that the short-term acidic stimulation could increase cell viability, as shown in Figure 3E. This also corresponded with a higher proliferation rate of hBMSCs, as determined by immunocytochemical staining for the proliferation marker, Ki-67, as shown in Figure 3C,D.

### 2.3. Short-Term Acidic Stimulation Enhances the Stem Cell Phenotype of MSCs

**Figure 4:**
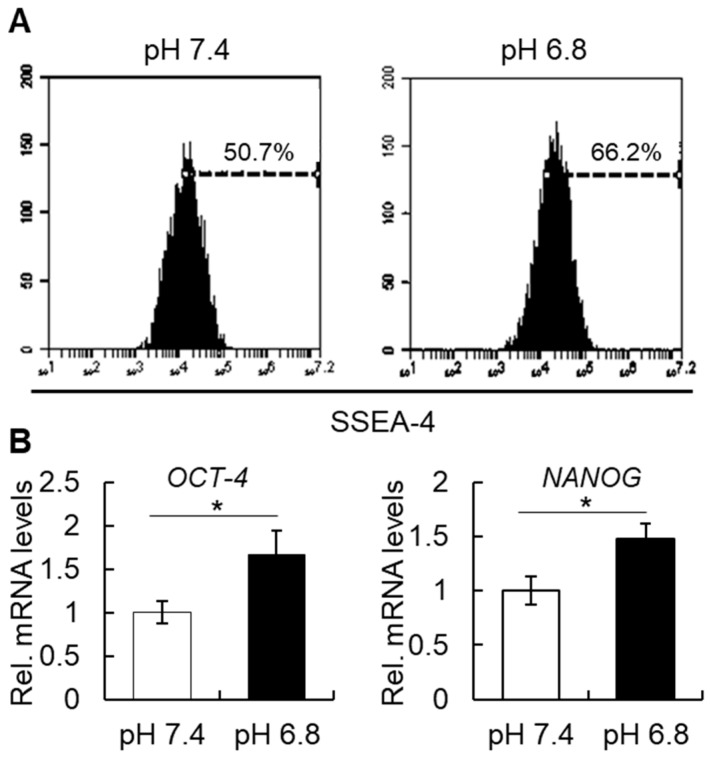
(**A**) Flow cytometric analysis of the expression of SSEA-4 in hBMSCs after short-term treatment with acidic pH. Note an increase in SSEA-4 expression levels in the cells cultured in pH 6.8. Images are representative of at least 3 independent experiments. (**B**) Relative mRNA expression levels of the early stem cell markers, *OCT-4* and *NANOG*, in hBMSCs. Note the higher expression of the two genes after short-term treatment in pH 6.8. The graph shows representative data (mean ± average) of 3 independent experiments. * *p* ≤ 0.05; Student’s *t*-test.

hBMSCs were cultured in acidic pH for two days and then submitted to analysis of the expression of stem cell markers. As shown in Figure 4A, the short-term acidic stimulation enhanced the expression of SSEA-4 in approximately 15% of the cells. Additionally, the mRNA levels of early stem cell markers, *OCT-4* and *NANOG*, also increased by a short-term stimulation with a pH, as shown in Figure 4B.

### 2.4. Short-Term Acidic Stimulation Decreases the Migration Ability of MSCs

**Figure 5:**
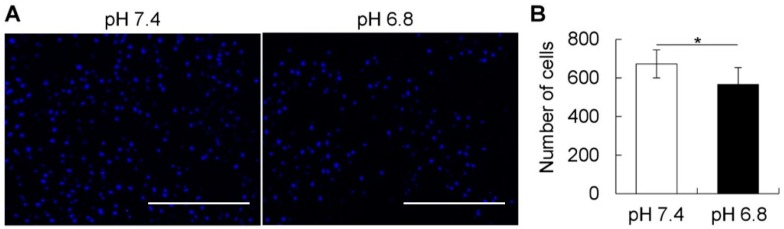
(**A**,**B**) Images and quantitative analysis of the number of migrated cells in the lower side of the Boyden chamber. Note the decrease in the migration ability of hBMSCs cultured temporarily in acidic condition (pH 6.8). Data are representative of at least 3 independent experiments. * *p* ≤ 0.05; Student’s *t*-test. Scale bars: 500 μm.

The Boyden chamber method was used to evaluate the migration ability of hBMSCs under different pH conditions. hBMSCs were plated in the upper chamber cultured in acidic pH for two days, and the cells that migrated towards the lower chamber were counted. As shown in Figure 5A,B, the short-term acidic stimulation suppressed the migration ability of hBMSCs.

## 3. Discussion

In this study, we first showed that MSCs appear at the granulation tissue of the bone healing site in the initial two days. In vivo measurement of pH variation showed a decrease up to pH 6.8 in the initial two days (inflammatory period). In fact, the measurement of pH during tissue healing has been a challenge [8]. In skin inflammation, previous studies detected pH levels within a range of 4.7 to 5.7 [9]. In bone tissue, reports on the measurement of pH are scarce. Only one previous study using a non-invasive method showed the pH in the initial stage of healing to be neutral to slightly acidic (pH 7.2). However, since it was non-invasive, the results could be just an estimation. In this study, the fiber optic pH sensor enabled a precise measurement of the changes in pH during the initial seven days of bone healing. To the authors’ knowledge, there is no other method currently available that allows the measurement of pH at a very small area, such as in the tooth extraction socket or femur defect of mice. Overall, the present data corroborate with previous findings showing an acidic period during the inflammatory stage of in vivo tissue healing.

The effect of acidic treatment on cells in vitro has been reported previously as a means to reproduce an inflammatory condition or to evaluate the effect of environmental pH on cellular functioning. A previous paper has demonstrated that a low pH treatment induces a significant increase in stem cell markers (*Oct4*, *Nanog*) in cancer stem cells [10]. Moreover, since the stem cell phenotype of cancer stem cells is known to be associated with their metastatic activity, it would be possible that cancer stem cells pre-treated in an acidic pH could show a higher metastatic activity. Indeed, a previous study showed that acidic pH promotes experimental pulmonary metastasis of human melanoma cells in athymic nude mice by up-regulating the expression of the proteolytic enzymes MMP-2, MMP-9, cathepsin B, and cathepsin L and the proangiogenic factors VEGF-A and IL-8 [11]. Another study investigated the effect of acidic pH on the proliferation and mineralization of BMSCs [12]. The report demonstrated that acidic treatment decreases proliferation and mineralization ability of BMSCs [12]. In pH below 6.5, it is in fact, less likely that hydroxyapatite is deposited [12]. The present study showed that a short-term treatment of hBMSCs in acidic pH significantly enhances their stem cell phenotype. Moreover, a lower pH of 6.4 induced a much more marked increase in the stem cell phenotype of hBMSCs, but that was followed also by a dramatic decrease in cell viability, as shown in Figure S1.

In addition to the effect of the external pH, inflammatory cytokines have also been shown to induce changes in the stem cell phenotype of somatic cells. Our group previously showed that a 2-day treatment with tumor necrosis factor-alpha (TNFα), but not with other inflammatory cytokines, such as interleukin-1 or 6, can induce a significant increase in the percentage of cells positive for SSEA-4, CD-146, STRO-1, as well as a significant increase in *OCT-4* and *NANOG* transcript levels [2]. Taken together, these data support the notion that an inflammatory condition, which includes a low pH and biological action of cytokines, can regulate the stemness of cells.

The increase in expression of stem cell markers in hBMSCs could reflect a higher potential of the cells to differentiate into other cell types, such as osteoblasts, chondrocytes, or adipocytes. Nevertheless, in vitro cell differentiation experiments showed that a 2-day pre-conditioning of hBMSCs did not affect their osteogenic or adipogenic ability (data not shown). It might be possible that the in vitro culture methods used to evaluate cell differentiation, which requires at least 14 to 21 days, would be inappropriate to investigate the effect of a short-term pH treatment on the cell differentiation ability of hBMSCs.

Regarding the morphometric changes in hBMSC shape, the effect of pH on the morphology of hBMSCs could be associated with a change in the difference between the outer and the inner monolayer areas of the cell membrane lipid bilayer, as described previously [13,14]. The cell membrane consists of a phospholipid bilayer and the underlying membrane skeleton, which is a complex network of interlinking filaments and tubules that extend from the nucleus throughout the cytoplasm, towards the plasma membrane. By taking into consideration that the stable cell shape corresponds to the minimum of the membrane energy, which consists of the bending and stretching energies of the bilayer and the skeleton and of the bilayer-skeleton interaction energy [13]. Thus, changes in environmental pH could be directly disturbing the minimum membrane energy, and induce cell shape change. In this study, the slight decrease in pH, from 7.4 to pH 6.8, did not dramatically affect the shape of hBMSCs. However, at pH 6.4, the cells presented a remarkable change, becoming notably thinner and smaller.

The present results also showed that after seven days of healing in the femur, there was a tendency of pH to increase towards the alkaline condition. In fact, previous studies have shown that an alkaline pH of 8.0 is optimal for mineral deposition by MSCs differentiated into osteoblasts [15]. In accordance with these findings, mineral deposition in vivo starts from day 7 of healing. However, due to the fragility of the instruments used herein, it was not feasible to directly measure the pH in vivo for a longer period of time, until complete tissue healing.

In summary, a short-term acidic stimulation enhanced the stem cell phenotype of hBMSCs, which could be helping to enhance the differentiation ability towards osteoblasts and as well as bone tissue healing. These findings may further help the development of materials and methods for more precise and on-site control of the stemness and function of BMSCs, which can also have implications in the optimization of conditions for BMSC transplantation towards immunotherapy and tissue healing.

## 4. Materials and Methods

### 4.1. Animal Experiment and pH Measurement

C57BL/6 mice were purchased from CLEA Japan (Tokyo, Japan), and maintained according to the Guidelines for Animal Research of Okayama University, under the approval of the Animal Care and Use Committee of Okayama University (OKU-2014283). For surgical defects in the mouse femur, a lateral incision in the medial side of the femur was made to open the quadriceps muscles, which was subsequently incised to open the femur. The surgical defect was performed with a steel round bur of 1 mm in diameter (Dentsply-Sirona, Ballaigues, Switzerland) using a micromotor and handpiece. Immediately after surgery, the defect was slightly washed with sterilized phosphate-buffer saline (PBS) to remove bone debris, and the muscle and skin were then returned to their original position and sutured. Measurement of pH at 0 h was performed immediately after the defect was drilled and after blood coagulation. Tooth extraction was performed according to methods described previously [3]. pH was measured immediately after, as well as 6 h, 12 h, 1 day, 2 days, 3 days, 5 days and 7 days after the surgical defect or tooth extraction. A total of 4 different bone wound healing sites at each time point (0 h, 6 h, 12 h, 1 day, 3 days, 5 days, and 7 days) were assessed for the in vivo pH measurement analysis.

For measurement of pH at the healing site, we used a needle-contained optical fiber (NTH-HP5, PreSens Precision Sensing GmbH, Regensburg, Germany) of 140 μm in diameter connected to a pH microsensor (pH-1 microsensor, PreSens Precision Sensing GmbH) with a resolution of ± 0.02 pH and accuracy of ± 0.1 pH (at pH = 7), and a pH measurement range from 5.5 to 8.5. The pH microsensor was connected to a computer, where all data was saved and analyzed.

The needle containing the optical fiber microsensor was placed into the granulation tissue of the healing bone defect or healing tooth extraction socket and kept in place for 60 s. The pH microsensor device was set to show an average of 4 measurements per second. The final pH at each time point was determined by the average of at least 20 pH measurements obtained from each of the four independent healing sites. In the case of the femur defect at day 7 only, the average was taken from 3 different healing sites. The microsensor was washed with distilled water after each measurement. Each site was only measured once. Calibration of the microsensor was performed before each measurement in a PBS solution titrated to pH 7.5.

### 4.2. Histological and Immunohistochemical Analysis

For the preparation of frozen sections from non-fixed and undecalcified hard tissues, Kawamoto’s film methods were used. Samples were freeze-embedded with super cryoembedding medium (SECTION-LAB Co. Ltd., Hiroshima, Japan) and cut in thickness of 5 μm after mounting the adhesive film onto the sample surface. Samples were then immediately fixed with 4% paraformaldehyde (PFA) for 20 min and stained with hematoxylin and eosin. For immunohistological analysis, the specimens were incubated with the anti-CD146 antibody (Abcam, San Francisco, CA, USA), or the isotype IgG (Abcam) at 4 °C overnight after blocking with 5% goat serum (Life Technologies, Gaithersburg, MD, USA). After washing, the specimens were incubated with secondary antibody Alexa Fluor 488 donkey anti-rabbit IgG (Life Technologies) for 60 min at room temperature. All images were taken by fluorescence microscope (Biozero BZ-X700, Keyence, Osaka, Japan).

### 4.3. Cells and Culture Conditions

hBMSCs were purchased from Lonza (Walkersville, MD, USA) and cultured in alpha-Modified Eagle Medium (Invitrogen, Carlsbad, CA, USA) containing 15% fetal bovine serum (FBS, Invitrogen), 100 mM L-ascorbic acid 2-phosphate (Wako Pure Chemical Industries, Osaka, Japan), 1% penicillin and streptomycin (Sigma), and 1% L-glutamine (Invitrogen). Media were titrated to pH 7.4, pH 6.8, or pH 6.4 by adding 20 mM HEPES and 1 M HCl, as described previously [10,16]. Media were retitrated after incubation for 24 h, before using in the experiments. Cells were then cultured under different pH conditions for 48 h, before analysis of the stem cell characteristics and functions.

### 4.4. Real-Time Reverse-Transcription Polymerase Chain Reaction (RT-PCR) Analysis

hBMSCs were cultured at pH 7.4 or pH 6.8 for 48 h, and total cellular RNA was extracted using Purelink (Life Technologies), according to the manufacturer’s instructions. To remove potential residual DNA the samples were treated with DNase I (DNASE I, Invitrogen). Real-time RT-PCR was used for mRNA quantitation as described [17,18]. The levels of mRNA of interest were normalized to that of the reference gene ribosomal protein S29. Primer sequences are shown in Table 1.

### 4.5. Cell Viability Assay

Cells were cultured in different pH for 2 days and then cell viability was estimated by MTS assay (CellTiter 96^®^ AQueous One Solution Cell Proliferation Assay; Promega, Madison, WI, USA), according to the manufacturer’s instructions.

### 4.6. Immunocytochemistry

For immunocytochemical analysis, hBMSCs were cultured under pH 7.4 or pH 6.8 for 48 h and then fixed with 4% PFA for 20 min, washed in PBS, blocked with 5% goat serum and then immunolabeled with primary antibody, or the isotype-matched IgG antibody. The target proteins were visualized with secondary antibody conjugated with Alexa Fluor 488 or 647 (Life Technologies) under a fluorescence microscope (Biozero BZ-X700, KEYENCE). Antibodies for Ki-67 was purchased from Abcam (Cambridge, UK).

### 4.7. Migration Assay

The migration assay was performed in the Boyden chamber using cell culture inserts with a light-opaque polyethylene terephthalate of 8 μm microporous membrane (BD Falcon HTS FluoroBlokTM inserts, BD Biosciences). hBMSCs were dissociated with Accutase (Innovative cell technologies, San Diego, CA, USA), counted and seeded in the upper chamber (cell insert), and incubated for 24 h in different pH conditions. The cells were fixed with 4% PFA and washed with PBS, before nuclei staining and observation under a fluorescent microscope (Biozero BZ-X700, KEYENCE). The total number of migrated cells observed at the bottom of the chamber were counted in four different pictures taken per chamber/insert. Images of the cells were captured using fluorescence microscopy (Biozero BZ-X700, KEYENCE) and further binarized and counted using ImageJ software (National Institutes of Health, Bethesda, MD, USA).

### 4.8. Flow Cytometry (FCM)

hBMSCs were cultured under pH 7.4 or pH 6.8 for 48 h, then dissociated with Accutase, filtered through a 70 μm cell strainer, washed, and resuspended in phosphate-buffered saline (PBS) containing 1% FBS at a concentration of 1 × 10^6^ cells per 100 µL. Cells were then incubated with anti-human SSEA-4 antibody (BD Biosciences) or isotype control (IgG) for 30 min on ice, washed, and subjected to FCM analysis by Accuri C6 (BD Biosciences) [2].

### 4.9. Statistical Analysis

Analysis of the differences between groups was performed with unpaired Student’s *t*-test, or one-way ANOVA followed by a Fisher’s post-hoc correction test when appropriate. Statview software (version 5.0; SAS Institute Inc., Cary, NC, USA) was used for the analyses.

## Figures and Tables

**Table 1:** Primer sequences used in real-time RT-PCR analysis.

Gene		Primer Sequence	PCR Product Length (bp)
*s29*	Sense	5′-TCTCGCTCTTGTCGTGTCTGTTC-3′	75
	Antisense	5′-ACACTGGCGGCACATATTGAGG-3′	
*OCT-4/POU5F1*	Sense	5′- CCGAGTGTGGTTCTGTAAC-3′	196
	Antisense	5′-GAAAGGGACCGAGGAGTA-3′	
*NANOG*	Sense	5′-TCTCCAACATCCTGAACCT-3′	117
	Antisense	5′-GCGTCACACCATTGGTAT-3′	

## Data Availability

All data are available upon request to the corresponding authors.

## Supplementary Materials

Supplementary materials can be found at https://www.mdpi.com/1422-0067/20/5/1097/s1. Figure S1: Effect of short-term treatment of pH 6.4 on stem cell properties of BMSCs.

## References

[1] Marsell R., Einhorn T.A. (2011). The biology of fracture healing. Injury.

[2] Ueda M., Fujisawa T., Ono M., Hara E.S., Pham H.T., Nakajima R., Sonoyama W., Kuboki T. (2014). A short-term treatment with tumor necrosis factor-alpha enhances stem cell phenotype of human dental pulp cells. Stem Cell Res. Ther..

[3] Nakajima R., Ono M., Hara E.S., Oida Y., Shinkawa S., Pham H.T., Akiyama K., Sonoyama W., Maekawa K., Kuboki T. (2014). Mesenchymal Stem/Progenitor Cell Isolation from Tooth Extraction Sockets. J. Dent. Res..

[4] Karp J.M., Leng Teo G.S. (2009). Mesenchymal Stem Cell Homing: The Devil Is in the Details. Cell Stem Cell.

[5] Eggenhofer E., Luk F., Dahlke M.H., Hoogduijn M.J. (2014). The life and fate of mesenchymal stem cells. Front. Immunol..

[6] Spector J.A., Mehrara B.J., Greenwald J.A., Saadeh P.B., Steinbrech D.S., Bouletreau P.J., Smith L.P., Longaker M.T. (2001). Osteoblast expression of vascular endothelial growth factor is modulated by the extracellular microenvironment. Am. J. Physiol. Cell Physiol..

[7] Newman R.J., Duthie R.B., Francis M.J. (1985). Nuclear magnetic resonance studies of fracture repair. Clin. Orthop. Relat. Res..

[8] Punnia-Moorthy A. (1987). Evaluation of pH changes in inflammation of the subcutaneous air pouch lining in the rat, induced by carrageenan, dextran and Staphylococcus aureus. J. Oral Pathol..

[9] Steen K.H., Steen A.E., Reeh P.W. (1995). A Dominant Role of Acid pH in Inflammatory Excitation and Sensitization of Nociceptors in Rat Skin, in vitro. J. Neurosci..

[10] Hjelmeland A.B., Wu Q., Heddleston J.M., Choudhary G.S., MacSwords J., Lathia J.D., McLendon R., Lindner D., Sloan A., Rich J.N. (2011). Acidic stress promotes a glioma stem cell phenotype. Cell Death Differ..

[11] Rofstad E.K., Mathiesen B., Kindem K., Galappathi K. (2006). Acidic extracellular pH promotes experimental metastasis of human melanoma cells in athymic nude mice. Cancer Res..

[12] Fliefel R., Popov C., Tröltzsch M., Kühnisch J., Ehrenfeld M., Otto S. (2016). Mesenchymal stem cell proliferation and mineralization but not osteogenic differentiation are strongly affected by extracellular pH. J. Cranio Maxillofac. Surg..

[13] Bobrowska-Hägerstrand M., Hägerstrand H., Iglic A. (1998). Membrane skeleton and red blood cell vesiculation at low pH. Biochim. Biophys. Acta.

[14] Iglic A., Svetina S., Zeks B. (1996). A role of membrane skeleton in discontinuous red blood cell shape transformations. Cell. Mol. Biol. Lett..

[15] Galow A.M., Rebl A., Koczan D., Bonk S.M., Baumann W., Gimsa J. (2017). Increased osteoblast viability at alkaline pH in vitro provides a new perspective on bone regeneration. Biochem. Biophys. Rep..

[16] Reichert M., Steinbach J.P., Supra P., Weller M. (2002). Modulation of growth and radiochemosensitivity of human malignant glioma cells by acidosis. Cancer.

[17] Hara E.S., Ono M., Pham H.T., Sonoyama W., Kubota S., Takigawa M., Matsumoto T., Young M.F., Olsen B.R., Kuboki T. (2015). Fluocinolone Acetonide Is a Potent Synergistic Factor of TGF-β3-Associated Chondrogenesis of Bone Marrow-Derived Mesenchymal Stem Cells for Articular Surface Regeneration. J. Bone Miner. Res..

[18] Hara E.S., Ono M., Yoshioka Y., Ueda J., Hazehara Y., Pham H.T., Matsumoto T., Kuboki T. (2016). Antagonistic Effects of Insulin and TGF-β3 during Chondrogenic Differentiation of Human BMSCs under a Minimal Amount of Factors. Cells Tissues Organs.

