# Supplementary material for: Acidic Pre-Conditioning Enhances the Stem Cell Phenotype of Human Bone Marrow Stem/Progenitor Cells
Source: Int J Mol Sci. 2019 Mar 4;20(5):1097. doi: 10.3390/ijms20051097 (PMC6429188; doi:10.3390/ijms20051097)
Supplement: Supplementary file 1 [file ijms-20-01097-s001.pdf]

International Journal of
*Molecular Sciences*

MDPI

# Supplemental Material

*Communication*

## Acidic Pre-Conditioning Enhances the Stem Cell Phenotype of Human Bone Marrow Stem/Progenitor Cells

Yuri Hazehara-Kunitomo [1], Emilio Satoshi Hara [2,]*, Mitsuaki Ono [3,]*, Kyaw Thu Aung [1], Keiko Komi [1], Hai Thanh Pham [1,4,5], Kentaro Akiyama [1], Masahiro Okada [2], Toshitaka Oohashi [3], Takuya Matsumoto [2] and Takuo Kuboki [1,]*

## Supplemental Figure S1

Effect of short-term treatment of pH 6.4 on stem cell properties of BMSCs.

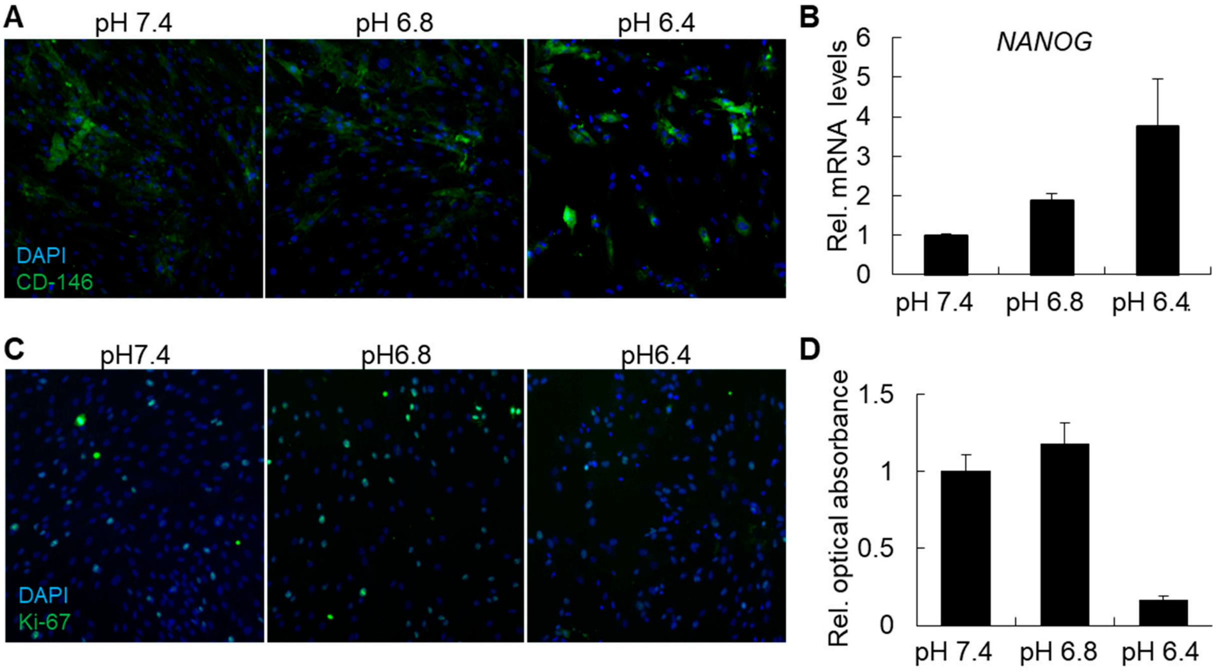


**Figure S1.** Immunocytochemical analysis for (**A**) expression of CD146 and (**B**) relative mRNA expression levels of *NANOG* in hBMSCs after a short-term treatment in pH 6.8 and pH 6.4. Note the substantial increase in stem cell markers in pH 6.4. Immunocytochemical analysis for (**C**) expression of the proliferation marker Ki-67, and (**D**) cell viability,

determined by MTS assay in hBMSCs after a short-term treatment in pH 6.8 and pH 6.4.

Note the dramatic decrease in cell proliferation ability and cell viability in pH 6.4.
